# Performance of yellow and pink oyster mushroom dyes in dye sensitized solar cell

**DOI:** 10.1038/s41598-024-73865-z

**Published:** 2024-10-10

**Authors:** Nur Alfarina Pirdaus, Nurfadzilah Ahmad, Nofri Yenita Dahlan, Ainur Nisha Redzuan, Aisyatul Husna Zalizan, Firdaus Muhammad-Sukki, Nurul Aini Bani, Muhamad Fazly Abdul Patah, Wan Abd Al Qadr Imad Wan-Mohtar

**Affiliations:** 1 Solar Research Institute (SRI), Universiti Teknologi MARA (UiTM), 40450 Shah Alam, Malaysia; 2 School of Electrical Engineering, College of Engineering, Universiti Teknologi MARA (UiTM), 40450 Shah Alam, Malaysia; 3 School of Computing, Engineering & the Built Environment, Merchiston Campus, Edinburgh Napier University, 10 Colinton Road, Edinburgh, EH10 5DT UK; 4 Smart Engineering and Advanced Technology Department, Faculty of Artificial Intelligence, Universiti Teknologi Malaysia, Jalan Sultan Yahya Petra, 54100 Kuala Lumpur, Malaysia; 5 Functional Omics and Bioprocess Development Laboratory, Institute of Biological Sciences, Faculty of Science, Universiti Malaya, 50603 Kuala Lumpur, Malaysia

**Keywords:** Dye-sensitized solar cells, Oyster mushroom dyes, Electrical properties, Optical properties, Structural properties, Current density-voltage (J-V) characteristics, Biomaterials, Solar energy, Photovoltaics, Solar cells

## Abstract

A solar photovoltaic (PV) cell, is an electrical device that uses the PV effect to convert light energy into electricity. The application of oyster mushroom dyes in dye sensitized solar cell (DSSC) is a novel strategy to substitute the costly chemical production process with easily extractable, environmentally acceptable dyes. Both dyes of yellow and pink oyster mushrooms were extracted using the same process but dried into powder form using two techniques, warm drying and freeze drying. The characterization was carried out utilizing current-voltage (I-V) characterization for electrical properties, Ultraviolet-Visible (UV-Vis) spectrophotometer for optical properties, Field Emission Scanning Electron Microscopy (FESEM), and Atomic Force Microscopy (AFM) for the structural properties. It was found that freeze-dried pink and yellow oyster mushroom had shown the good properties for DSSC application as it produced energy bandgap which lies within the range of efficient dye sensitizer; 1.7 eV and 2.2 eV, the most uniform distribution of pores and a nearly spherical form in FESEM analysis, and AFM result obtained with the highest root mean square (RMS) roughness value (26.922 and 34.033) with stereoscopic morphologies. The data proved that mushroom dyes can be incorporated in DSSC with the optimization of drying method in the extraction process, dilution of dye and the layer of deposition on the glass substrate. The current density-voltage (J–V) characteristics of fabricated DSSC was characterized using Newport Oriel Sol3A solar simulator under AM 1.5 Sun condition (100 mW/cm^2^, 25 ^o^C). From the result obtained by solar simulator, the fabricated FTO/TiO_2_/*Pleurotus djamor* dye/Pt indicated the *V*_*oc*_ of 0.499 V and *J*_*sc*_ of 0.397 mA/cm^2^.

## Introduction

The third-generation solar cells such as hot carrier cells, polymer solar cells, quantum dot solar cells, dye-sensitized solar cells (DSSCs) and heterojunction cells prioritize efficiency optimization and production cost reduction^1^. Due to their ability to convert energy at a low cost, DSSC have sparked interest in photovoltaic technology. The use of naturally occurring, low-cost, non-toxic, and environmentally friendly pigments may eventually replace costly chemical synthesis techniques^2^. DSSCs have been the subject of extensive research over the last few decades due to their low manufacturing cost, ease of fabrication^3^, and relatively rising photo-conversion efficiency. Counter electrode, electrolyte solution with reduction-oxidation process, and dye-sensitized semiconductor photoanodes are the components of DSSCs^4^.

During the operation of a dye-sensitized solar cell (DSSC), when the cell is exposed to light, an incident photon is absorbed by a dye molecule and transition to an excited state. The dye molecule, when in an excited state, introduces an electron to the conduction band boundaries of the semiconductor. The regeneration of oxidized dye molecules is facilitated by the reduced redox species I^-^, which restores the dye to its original state and enables it to absorb an additional photon. The utilization of nanostructured semiconductor in photoelectrode films can greatly improve the performance of solar cells. This is achieved by providing a substantial surface area for the adsorption of dyes, direct pathways for the transport of photoexcited electrons, and efficient scattering centers that enhance the efficiency of light harvesting^5^. In a conventional DSSC, a layer of semiconducting metal-oxide is present, serving as a platform for dye anchorage and acting as a charge carrier through which dye is transported to the circuit^6^.

Currently, the most impressive power conversion efficiency (PCE) of DSSC obtained was 14.7% and achieved through the study of co-sensitization of a carbazole-based dye and a triphenylamine dye^7^. Other than that, the photovoltaic performance of TiO_2_-based DSSCs sensitized with anthocyanin and chlorophyll obtained a PCE of 0.66% and 0.528% respectively^8^. A study of DSSC incorporated with natural dyes extracted from mangosteen pericarp has produced a PCE of 1.17% while yellow and blue dyes extracted from *Gardenia Jasminoide Ellis* dyes has produced a PCE of 0.59%^9^. The PCE obtained for DSSC utilizing aloe vera, false daisy and *carica papaya* dyes were 0.75%, 0.3% and 0.39% respectively^10^. Meanwhile, the study which used red spinach as the dye for DSSC obtained the PCE of approximately around 0.032–0.148%^11^.

The photosensitizer dye is an essential element of the DSSC process. The efficiency of the cell is significantly influenced by the absorption spectrum of the dye and its level of anchoring to the semiconductor surface. The chemical composition of dyes plays a crucial role in enhancing photon absorption, hence leading to improved efficiency in electron injection and conversion^12^. To ensure the successful transfer of electrons from dyes to the conduction band of a semiconductor, an optimal sensitizer for DSSC must possess the following characteristics: (i) a strong binding affinity to the semiconductor through an anchoring group, such as a carboxylic or hydroxyl group; (ii) a significant absorbance in the visible and near-infrared parts of the solar spectrum, leading to a high molar absorbance coefficient; and (iii) the ability to facilitate the transfer of electrons from the dye. In order to facilitate charge injection into the semiconductor, it is necessary for the energy level of the lowest unoccupied molecular orbital (LUMO) to exceed that of the conduction band, while the energy level of the highest occupied molecular orbital (HOMO) must be lower than that of the redox pair. Consequently, the process of oxidizing dye renewal can be achieved^13^.

The Ru(II) and Os(II) polypyridine complexes are the most effective and commonly used dye sensitizers in the DSSC. However, it should be noted that these metal complex dyes are costly, and their manufacture necessitates a series of intricate and laborious procedures. The use of metal-free organic dyes was explored as a potential alternative to expensive metal complex dye sensitizers in DSSC. Researchers conducted investigations on several organic and inorganic dyes for the purpose of sensitization in DSSCs. Ruthenium compounds containing terpyridine moieties have shown to be highly effective sensitizers due to their wide spectrum of light absorption capabilities. These dyes possess the ability to absorb light within the near-infrared spectrum^14^. Nevertheless, ruthenium polypyridyl complexes are composed of heavy metals that have the potential to do harm to the environment. This necessitates the development of a natural dye with sensitizing capabilities by researchers^15^.

In recent years, interest in the use of natural dyes as sensitizers in DSSC has increased substantially due to their low cost and simple production. An extensive array of plant components may be utilized to derive natural dyes, such as fruits, seeds, leaves, stems, roots, and flowers^16^. Natural pigments are composed of chemical compounds that are vital to the development of all living things. On the basis of their biosynthetic basis and similar structure, plant pigments are classified as chlorophylls, carotenoids, anthocyanins, or flavonoids. Natural dyes are less stable and less efficient than synthetic dyes, which are uncommon, costly, and composed of potentially hazardous substances. Conversely, natural dyes possess a multitude of benefits in comparison to synthetic dyes, encompassing cost-effectiveness, accessibility, ecological compatibility, and biocompatibility^17^.

The effectiveness of natural DSSCs is impacted by several factors. These include the selection of an appropriate natural dye, the dye’s ability to provide stable, quantitative electronic anchoring to the solvent system, the photoanode, counter electrodes, and metal oxide nanostructure surfaces. In light of this, standardization of these critical components becomes critical with regard to the commercialization of natural DSSCs. Natural dyes are inexpensive, resource-limitless, environmentally friendly, possess high absorption coefficients, optimize light harvesting, and have no resource restrictions; their production is also straightforward. Despite the fact that the efficacy of these natural dyes remains insufficient for large-scale practical applications, this has not deterred scientists from around the globe from their pursuit of discovering new natural dye sources. Ongoing are these captivating inquiries into the production of DSSCs using dyes obtained from bioresources^18^.

Twenty years ago, the scientific community began to discuss the use of natural dyes as sensitizers for DSSCs; however, the results have been unsatisfactory in terms of efficacy. Multiple studies have uncovered efficiencies below 2%. A subset of them has achieved a PCE of 5%. Natural dyes are suitable sensitizers for DSSCs due to their extensive availability, ecology compatibility, economic viability, and biodegradability. However, prior records indicate that no one reported a PCE exceeding 10% for natural dyes. A significant proportion of them possess PCE levels ranging from 3 to 4%. One limitation of natural dyes is their inherent instability. Additionally, they aggregate readily, rendering the collection of photogenerated charges ineffective. The vast majority of them exhibit inadequate adhesion to the oxide semiconductor surface. The lack of research on alternative dyes presents an opportunity for further investigation, as the derivatives of this dye have not garnered significant attention^19^.

Unlike plant-based dyes, while being non-photosynthetic, fungi and their pigments have not conventionally been used as sensitizers. However, these organisms produce a number of secondary metabolites, including melanin and carotenoids, which have the potential to be used as natural sensitizers in DSSC. Fungal pigments are known for their wide pH and temperature stability, and despite the fact that they do not possess light-harvesting functions, a thorough analysis of structure and anchoring groups can provide some favorable pigment choices^20^. Mushrooms as a type of commercial fungal, are mostly colored and have various pigments identified in their species, such as carotenoid in *Cordyceps militaris* and the red pigment lilacinone in *Lactarius lilacinus*. Previous research found melanin in mushrooms such as *Auricularia auricula*, *Pleurotus cystidiosus*, *Armillaria cepistipes*, *Agaricus bisporus*, and *Lentinula edodes*. Melanin is a group of high-weight molecules composed of complex heterogeneous polymers of phenolic and/or indolic monomers that exist in three main forms: eumelanin, pheomelanin, and allomelanin^21^. Due to these benefits of fungal or specifically mushroom dye, oyster mushroom was utilized as photosensitizer for DSSC in this work.

The novelty of the present work is the construction of DSSCs from the natural dye from the optimized oyster mushroom dye. In this work, the extracted dyes from two types of oyster mushroom, pink (*Pleurotus djamor*) and yellow (*Pleurotus citrinopileatus*) with different drying method, dilution ratio of dye powder to deionized (DI) water and deposition thickness are presented for optimization purpose in DSSC application. The electrical, optical, physical, and chemical behaviors of the dyes are characterized by I-V test, UV-Vis-NIR Spectroscopy, Atomic Force Microscopy (AFM), Field Emission Scanning Electron Microscopy (FESEM), and FT-IR Spectroscopy while the device efficiency is measured using solar simulator.

## Materials and methods

### Oyster mushroom dye isolation and purification

**Fig. 1:**
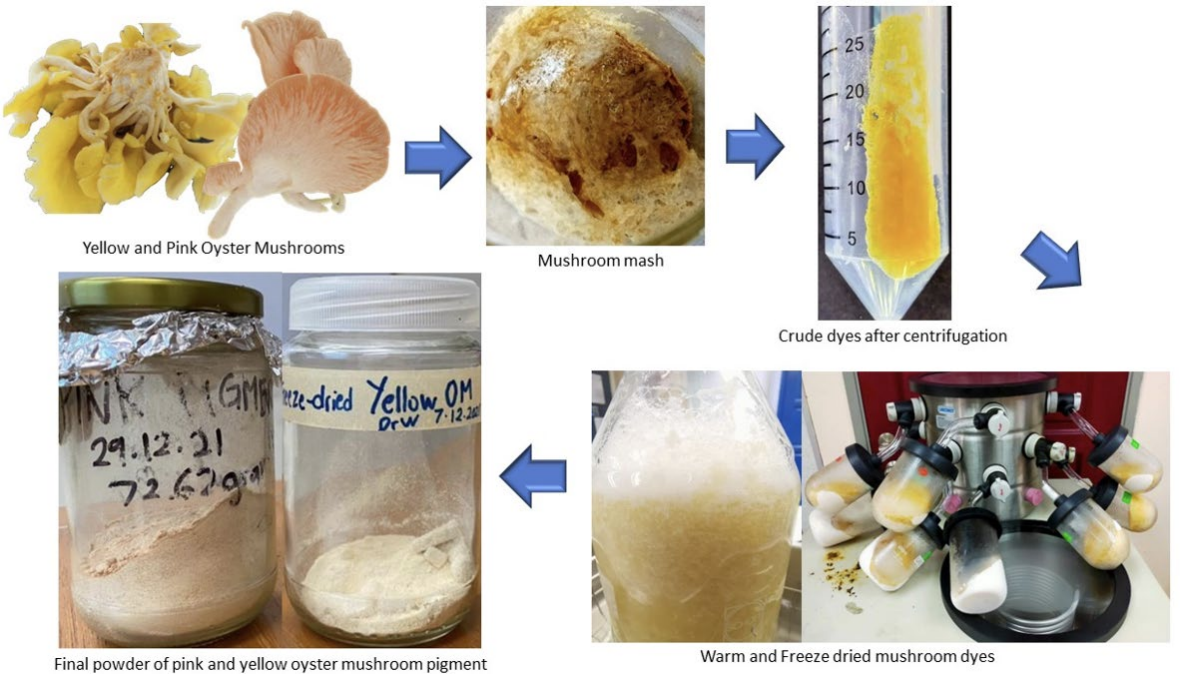
Process flow of the oyster mushroom dye isolation and purification.

The isolation and purification fungal pigments from the oyster mushrooms performed using the procedure that has been described in the literature with a minor adjustment^21^. Figure 1 summarizes the process flow of the oyster mushroom dye isolation and purification carried out in this paper.

A homogenizer was used to crush the fruiting bodies of the yellow (*Pleurotus citrinopileatus*) and pink (*Pleurotus djamor*) mushrooms. The mushroom mash and sodium hydroxide solution were then suspended together in a volume ratio of 1:30. To ensure complete extraction, the suspension was incubated in 80 W sonicator (Fisher Scientific, USA) for 80 min. Centrifugation was be used to separate the residual material for 15 minat 10,000 rpm. The pH of the supernatant was then lowered to 1.5 by adding a 7 mol/L solution of hydrochloric acid (HCl) in an Erlenmeyer flask. At room temperature, the supernatant was allowed to precipitate for three hours.

The precipitate was recovered and rinsed with deionized water until its pH was neutral after being centrifuged at 10,000 rpm for 15 min. In a freeze dryer, the unprocessed pigments were recovered and dried in freeze dryer (Delta 1–24 LSC, Germany) for freeze drying and food dehydrator for warm drying respectively^21^.

Three different oyster mushrooms dyes which are pink freeze-dried oyster mushroom dye (PFOM), yellow freeze-dried oyster mushroom dye (YFOM) and yellow warm-dried oyster mushroom dye (YWOM) were ready.

### Spin coating process

Spin coat process was used to create thin films for atomic force microscopy (AFM) analysis purpose in the Nano-Electronic Center Laboratory (NET), Universiti Teknologi MARA (UiTM), Shah Alam, Malaysia. Spin coating is a process that uses centrifugal force to spread uniform thin layers on the surface of a substrate. Three oyster mushroom dyes were utilized as the substrates. Each type of substrates was coated for 1 layer, 2 layers and 3 layers. Each layer contains 10 drops of the mushroom extract. The spin coater was set to 3,000 rpm for 30 s. The furnace was set to 100 °C for 10 min.

### Preparation of TiO2 thin film (anode)

The reagents used were all analytical grade, and they have not been further purified. To create the sol-gel synthesized TiO_2_, 10 ml of titanium (IV) butoxide was dissolved in absolute 10 ml of ethanol and agitated for an hour. 5 ml of distilled water was then be gradually dropwise added to the solution. Nearly immediately, the ensuing gel was created, and it was stirred for a few minutes. The solution was be rapidly stirred for an hour to generate white colloidal precipitate. After 24 h, the solutions were filtered and further dried in the oven at 100 °C for 12 h. After drying, the material will be milled into a fine powder using ball mill machine. 2 g of the prepared TiO_2_ powder was added into 100 ml of ethyl alcohol and stirred for 30 min to form a homogenous TiO_2_ paste. Before usage, the solution was kept in the dark. 10 drops of TiO_2_ paste were dropped onto the dried and cleaned glass substrates at a speed of 3,000 rpm to create a single layer of coating. The glass substrate was then dried at 100 °C for 10 min. The same technique was repeated for second and third layer of coating. The final coating underwent a 30-min annealing at 500 °C.

The FTO/TiO_2_ photoanode were sensitized in a petri dish using an aqueous mushroom dye solution overnight. The mushroom dyes prepared earlier which stored in powder form will be diluted with deionized water prior to use. The optimized ratio of dilution was utilized in this step.

### Preparation of graphite coated counter electrode (cathode) and electrolyte

In order to make the counter electrode (cathode) for the experiment, uncoated FTO glass was coated with platinum paste on the conductive side.

0.1 g of iodine and 0.5 g of potassium iodide (KI) were added into 50 ml of acetonitrile. The solution will be agitated for 30 min and then stored in a closed bottle prior to use.

### Assembly of DSSC

Before they can be assembled together, the dye-sensitized solar cells were fixed to a TiO_2_ electrode cast with electrolyte and a platinum counter electrode. The two electrodes (anode and cathode were sandwiched together on the coated sides with binder clips. 3 drops of electrolyte were dropped in between the electrodes prior to analysis.

### Characterization

#### Current–voltage measurement

2-point probe was used to measure the current-voltage for electrical properties analysis. A metal contact of gold (Au) 99.9% with 60 nm thickness was deposited on TiO_2_/oyster mushroom dye photoanode. The deposition was conducted using thermal evaporator.

#### UV–Vis-NIR spectrophotometer

Optical properties of the mushroom pigments were evaluated using a Jasco/V-670 UV–Vis-NIR Spectrophotometer. The absorption coefficient (*α*), which is a measurement of how far a light beam of a specific wavelength or energy may travel through a thin film before it is absorbed by that film, was calculated using the optical transmittance spectra of the film and the film thickness data. Equation (1) was used to get the Lambert’s Law of a-C thin films^22,23^:

1 $$\alpha=(\,1/t){\text{ }}ln(1/T)$$

where *t* is the thickness of the film and *T* is its transmittance. The optical band gap is a unit used to describe the separation between the stretched state in the valence band and the conduction band. Equation 2 uses the Tauc connection to derive the optical band gaps (*E*_g_) for amorphous semiconductors^24^,

2 $${(\alpha h \nu)^\gamma}={\text{ }}A({E_g}-h\nu)$$

where *hv* denotes photon energy, *A* the Tauc parameter, *γ* denotes the nature of the electronic transition and *α* absorption coefficient. The optical gap is determined by the point at where the Tauc’s slope intersects the photon energy axis^25^.

The mushroom dye powder was diluted in five different dilution ratios of 1:6, 1:8, 1:10, 1:12 and 1:14 to form the dye solution. The spectrophotometer was used to measure the absorption properties of the oyster mushroom pigments solution in the wavelength ranges. Different wavelengths are produced by varying the concentration of mushroom dye solution. The deionized water served as the baseline^26^.

#### Field emission scanning electron microscopy (FESEM)

The measurement of the surface morphology was carried out using Field Emission Scanning Electron Microscopy (FESEM) to capture the microstructure image of the oyster mushrooms. The samples were prepared in powder form for FESEM. A FESEM micrograph was created using a JEOL JSN-7600F Field Emission Scanning Electron Microscope with a 100 kX magnification and a 10 kV electron high tension (EHT). For results comparison, the FESEM was magnified by 5000×, 10,000×, 30,000×, and 50,000×. FESEM provides topographical and elemental information with almost limitless depth of field at magnifications ranging from 10× to 300,000×. FESEM analysis of the samples will be used to determine the topography characteristics^27^.

#### Atomic force microscopy (AFM)

The surface morphology was investigated using the Park System XE-100 Atomic Force Microscope. The Atomic Force Microscope’s main component is a cantilever with a pointed tip^28^. A cantilever, which is typically constructed of silicon or silicon nitride, is used to scan the specimen surface. The forces between the tip and the sample cause the cantilever to deflect when they are brought together. The surface morphology of the sample will then become visible, and the scanning size area can be varied depending on the circumstances. Atomic Force Microscopy (AFM) is a technique for investigating the surface of a hard material at the atomic level. AFM generates 3-D surface pictures and uses a mechanical probe to magnify surface features up to 100,000,000 times.

#### Solar simulator

Electrical parameters including fill factor (*FF*), efficiency (*η*), short circuit current density (*J*_*sc*_), short circuit current (*I*_*sc*_), open-circuit voltage (*V*_*oc*_), the maximum power (*I*_*max*_), the maximum voltage (*V*_*max*_) and the maximum power (*P*_*max*_) were obtained using the solar simulator.

## Results and discussion

### Optical analysis of the oyster mushroom dye pigment

The transmittance and absorbance of pink and yellow oyster mushroom dyes were evaluated in the wavelength ranging from 300 to 800 nm using a Perkin Elmer (LAMBDA 750) UV–Vis-NIR Spectrophotometer.

UV–Vis-NIR absorption spectrum of 1:6, 1:8, 1:10, 1:12 and 1:14 dilution ratio of 3 oyster mushroom samples; (a) pink freeze-dried oyster mushroom (PFOM) dye (b) yellow freeze-dried oyster mushroom (YFOM) dye (c) yellow warm-dried oyster mushroom (YWOM) dye.

**Fig. 2:**
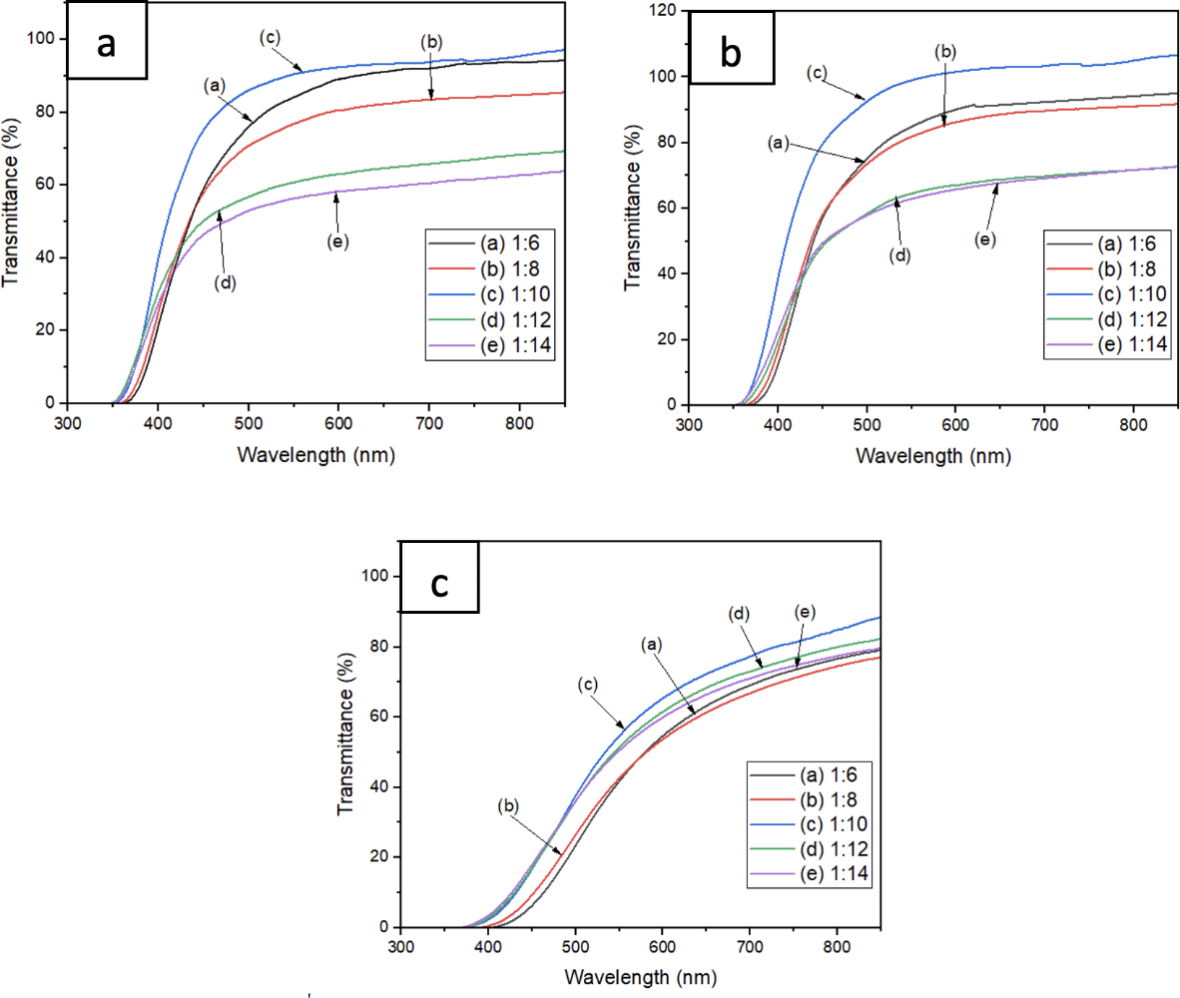
UV–Vis transmittance spectrum of 1:6, 1:8, 1:10, 1:12 and 1:14 dilution ratio of 3 oyster mushroom samples (**a**) pink freeze-dried oyster mushroom (PFOM) dye; (**b**) yellow freeze-dried oyster mushroom (YFOM) dye, and (**c**) yellow warm-dried oyster mushroom (YWOM) dye.

Figure 2 demonstrate the transmittance spectra of oyster mushroom dyes which all three dyes displayed the same continuous increasing trend of transmittance as the wavelength increased. High continuous transmittance (above 85%) is found at PFOM (Fig. 2a) and YFOM dyes (Fig. 2b) compared to YWOM dye (Fig. 2c). The low transmittance value of Fig. 2c in this study is predicted to be caused by excessive absorption. It has been established that the absorption coefficient is stronger at lower wavelengths than at higher wavelengths^25^. Visible light can travel through the photovoltaic without being absorbed due to its lower photon energy^29^. From the spectra at the visible range of 450–800 nm, having a high optical transmittance (> 85%) which in accordance with the solar spectrum, comprises the highest energy. In order to achieve high efficiency, the sample must have a high transmittance since energy must be transferred from one particle to another inside the sample^30^.

For PFOM and YFOM dyes, dye with 1:10 has the highest transmittance (> 80% starting) followed by 1:6, 1:8, 1:12 and 1:14. For YWOM dye, 1:10 ratio of dye dilution still has the highest transmittance among other four dilution but the highest transmittance increasing slowly and only reached more than 80% transmittance after 800 nm wavelength. The amount of light that the dye absorbs increases with dye concentration. This is due to the fact that a greater concentration of dye molecules offers more chances for light absorption, which modifies the transmittance spectrum.

However, self-quenching might occur at highly concentrated dye concentrations. This happens when molecules of dye are in close range to one another, resulting in interactions that can reduce the efficiency of electron injection and energy transfer processes. Very highly concentrated dye may cause dye aggregation at times which affecting the homogeneity of the dye layer. Aggregation can alter the optical properties of the dye and influence the transmittance spectrum. Dye aggregation is rather considered as an undesirable phenomenon in DSSC, leading to a reduced injection yield and lowered conversion efficiency by virtue of intermolecular excited state quenching^31^. Dye concentration optimization play crucial role in achieving better alignment between the absorption peaks of the dye and the solar spectrum, improving the overall efficiency of the solar cell.

**Fig. 3:**
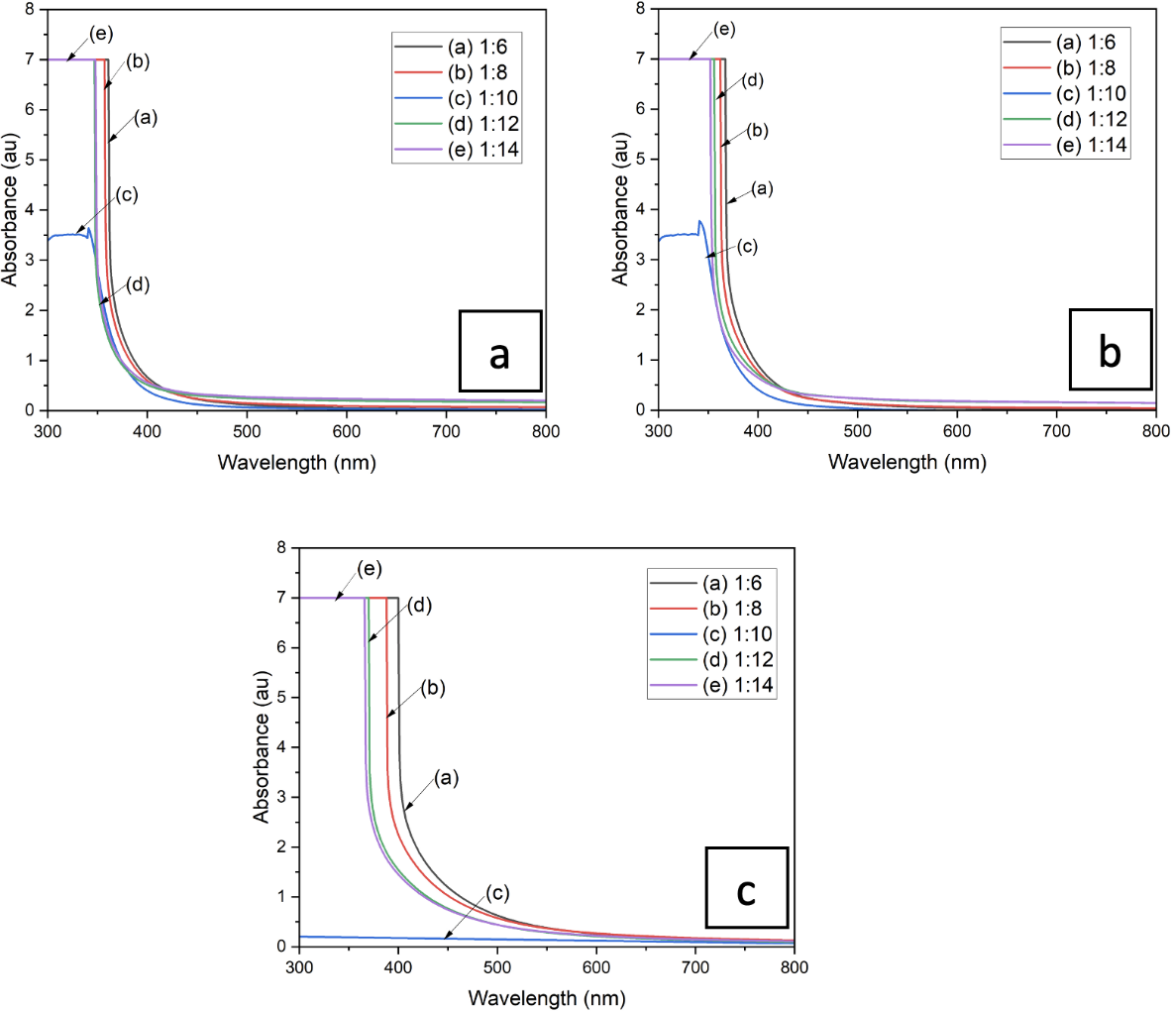
UV–Vis-NIR absorption spectrum of 1:6, 1:8, 1:10, 1:12 and 1:14 dilution ratio of 3 oyster mushroom samples (**a**) pink freeze-dried oyster mushroom (PFOM) dye; (**b**) yellow freeze-dried oyster mushroom (YFOM) dye, and (**c**) yellow warm-dried oyster mushroom (YWOM) dye.

Referring to Fig. 3, the extracted pigment of PFOM, YFOM and YWOM dyes show maximum absorbance within the UV region in the range of 300–400 nm. Previous study^32^ indicate that melanin was obtained from an *Actinoalloteichus sp*. strain, a type of bacteria strain with peak absorbance at 300 nm, tally with the results obtained.

Theoretically, maximum absorbance peaks of melanin are inconstant due to different existing types^32^. In this case, the maximum absorbance obtained for four dilutions (1:6, 1:8, 1:12, 1:14) which was 7 au, except for 1:10, which obtained an absorbance of 3.5 au. The absorbance spectra obtained also show that the values of absorption increased slowly from 200 to 280 nm and then increased intensively at 280 nm and become constant until 370 nm. The absorbance spectrum then decreased towards the visible region. This might be due to the presence of conjugated structures, which is a characteristic of pigments such as melanin^32,33^. It shows that oyster mushrooms have practically had their melanin extracted. This pattern can be seen in both PFOM and YFOM dyes but slightly different for YWOM dye. YWOM dye has longer wavelength with constant absorbance value. The absorbance spectra demonstrate that it could be used as dye material for DSSC. Lowering absorbance in peak of 1:10 for PFOM, YFOM and YWOM dyes expressed that this dilution ratio shows the most optimum absorbance. This dye acts as a sensitizer because the presence of dye in the film can bind with TiO_2_ and is expected to absorb more visible light types from the sun that comes when it is illuminated. As more light is absorbed, more electrons are transferred from the LUMO level to the TiO_2_ conduction band. This increases the number of electron transfers, thereby enhancing the efficiency of the solar cells^34^.

To support this finding, FTIR analysis of three oyster mushroom dyes were done and the results shown PFOM, YFOM and YWOM dyes representing mainly similar characteristic of transmittance peaks. Strong and broad characteristic absorption peak at around 3,280 cm^− 1^ indicating the presence of associated or polymeric –OH groups or N–H stretching vibrations of the carboxylic acid and phenolic groups of melanin^27,35^. The characteristic of strong band at 1,625 cm^− 1^ can be attributed to vibrations of aromatic ring C=C of amide I, C=O and/or of COO– groups^35^. The oyster mushroom dyes also exhibited characteristic bands at about 1,635 cm^−1^ (C–O stretching or aromatic C–C stretching), 1,398 cm^− 1^ (CN stretching)^27^.

The presence of O–H group and C–O groups play a major role in adsorption of dye onto the TiO_2_ surface. To enhance the electron transportation rate, the adsorption of the sensitizing dye to the TiO_2_ surface should be high. For the effective adsorption, the dye molecules should possess some functional group, which leads to the photon to electron conversion rate. It was mentioned in the previous report, presence of functional groups such as hydroxyl (–OH), esters and carbonyl (C–O) in the dyes are responsible for chemical adsorption of dye on the surface of nanostructured TiO_2_ film^16^. An efficient photosensitizer must possess several O or –OH groups capable of chelating to the Ti(IV) sites on the TiO_2_ surface^36^. High chemical adsorption of the dyes by the condensation of anchoring groups such as hydroxyl and methoxy protons with the hydroxyl groups on the surface of photoelectrode will enhance the dye performance as photosensitizer in DSSC^37^.

**Fig. 4:**
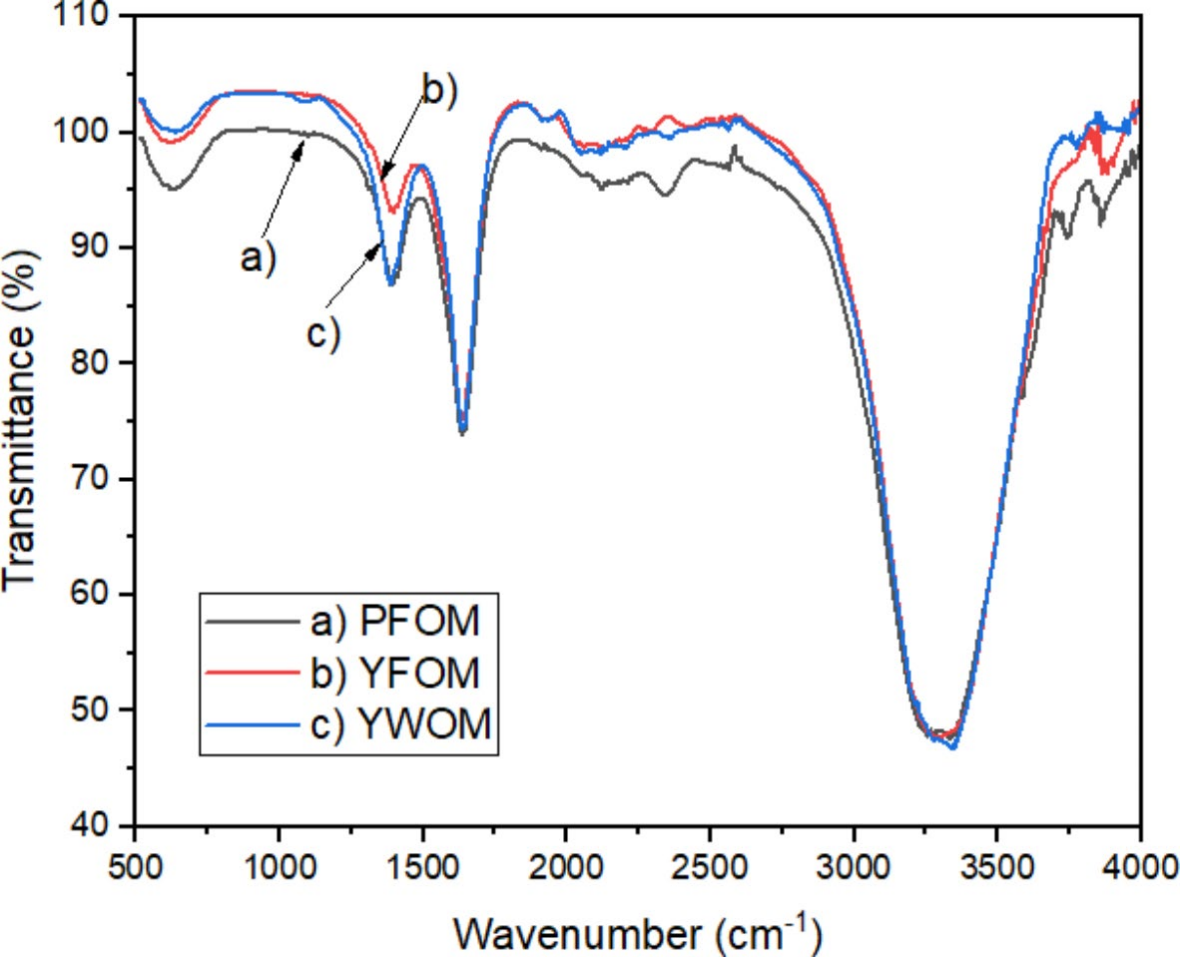
FTIR spectrum of 3 oyster mushroom samples; (**a**) pink freeze-dried oyster mushroom (PFOM) dye (**b**) yellow freeze-dried oyster mushroom dye (YFOM) (**c**) yellow warm-dried oyster mushroom dye (YWOM).

These FTIR results also supported the absorbance analysis in Fig. 4 indicating the presence of melanin pigment in the mushroom dye. Melanin has the unique ability to absorb almost every wavelength of light and in contrast to most other natural chromophores, has a broadband monotonic absorption spectrum^38^. The differences in maximum absorbance might be a result of the difference of melanin source causing minor change of the natural melanin structure^21^.

**Fig. 5:**
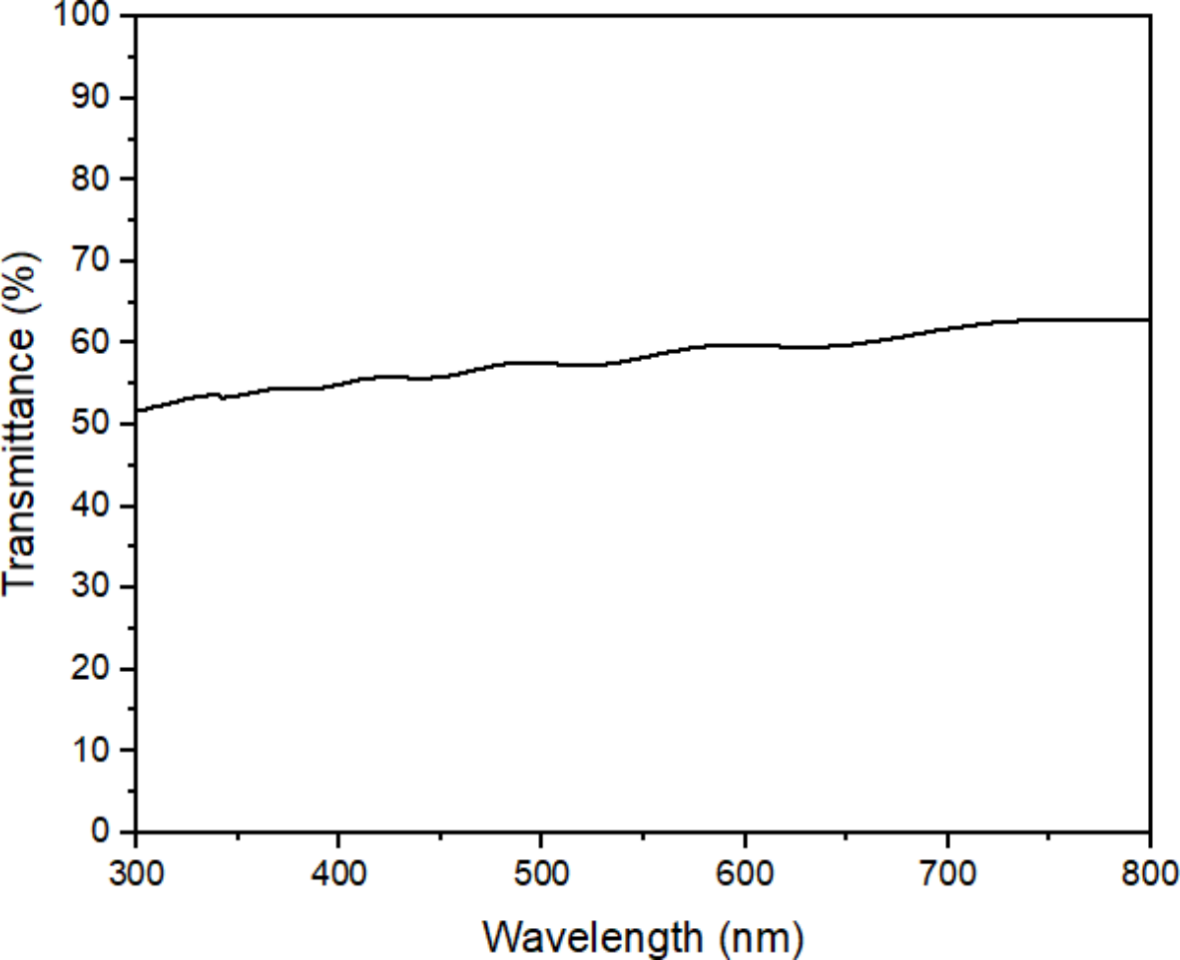
Transmittance spectrum of pink freeze-dried oyster mushroom (PFOM) dye with TiO_2_ coated on FTO glass (FTO/TiO_2_/PFOM).

The optical properties of the FTO/TiO_2_/PFOM were also studied using UV–Vis Spectrophotometer similarly with the individual dye. The optical transmittance spectra for FTO/TiO_2_/PFOM are shown in Fig. 5. The transmittance of the FTO/TiO_2_/PFOM shows only small increase of transmittance from 50 to 60% in the range of wavelength 300 to 800 nm. The transmittance is still considered low for TiO_2_-based solar cell (< 80%).

**Fig. 6:**
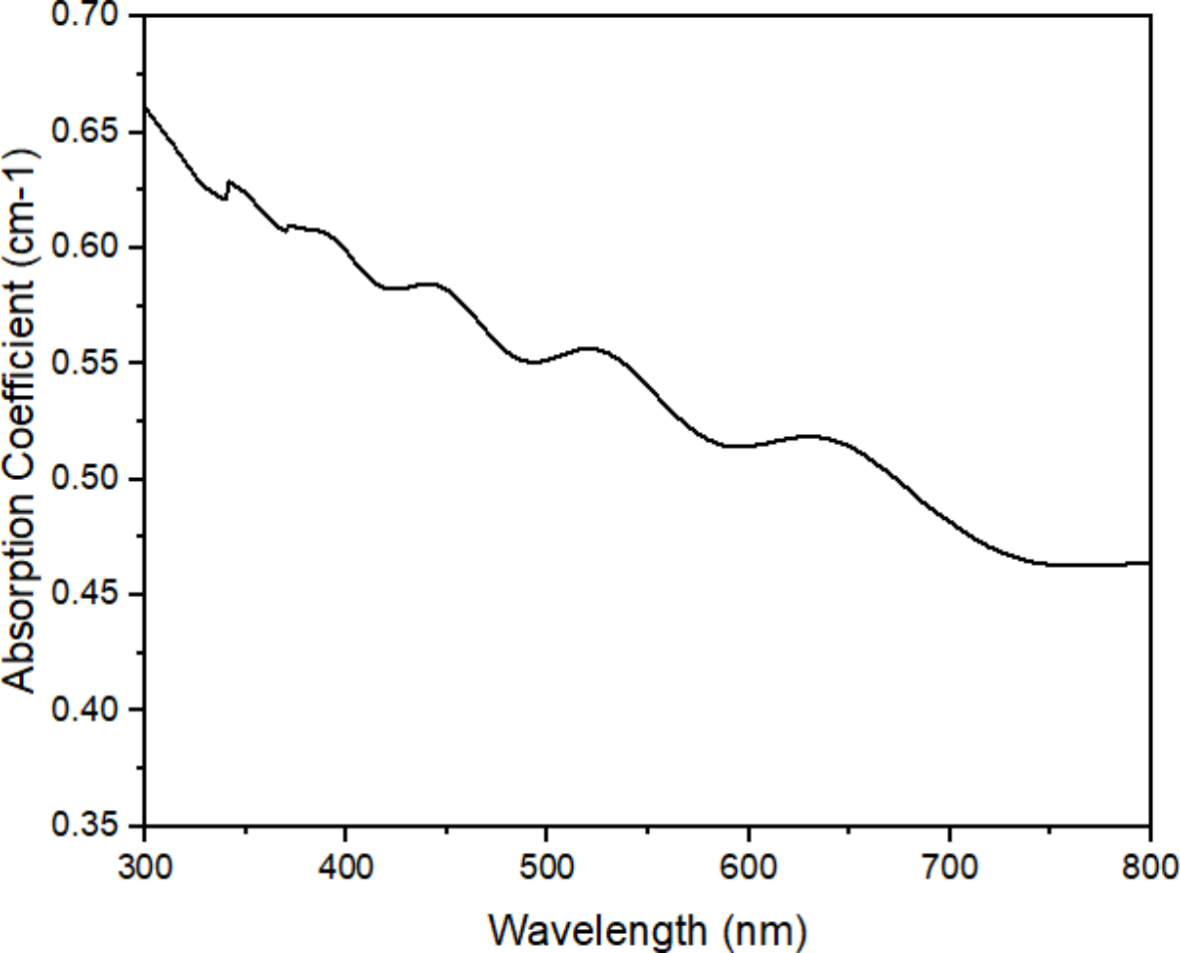
Absorption coefficient spectrum pink freeze-dried oyster mushroom (PFOM) dye with TiO_2_ coated on FTO glass (FTO/TiO_2_/PFOM).

Figure 6 shows the absorption coefficient spectra after sensitizing the PFOM dye on the TiO_2_ photoanode (FTO/TiO_2_/PFOM). The absorbance rate of individual oyster mushroom dye is in UV region of the spectrum is increased but in the case of FTO/TiO_2_/PFOM, several peaks can be seen from 300 to 800 nm wavelength with decreasing absorption coefficient values. FTO/TiO_2_/PFOM had made quite lower absorbance in the UV region and keep decreasing towards the visible region of the spectrum. This might be due to the excess dye molecule present during sensitization, such that excess dye molecule binds on top of another dye molecule on the layer of TiO_2_ surface. The binding strength of the aggregated complex might be too strong to break, and this condition may contribute to the lower photovoltaic function as it could not interact with the TiO_2_ surface for proper electron injection and poor interactions between the dye molecules and TiO_2_ molecule^16^.

Strong absorption abilities in the UV region are most probably caused by the presence of many UV active compounds. The strong visible light absorption abilities may have negative influence on the performance of DSSC sensitized by this dye. Theoretically, the dyes whose molecules have anchoring groups such as –COOH, –PO_3_H_2_, –SO_3_H, –OH, may be strongly bonded to the semiconductor surface and give effective sensitization^39^. Although –COOH and –OH are present in the oyster mushroom dye pigment, the occurrence of strong absorption in the UV range might cause the reduced in the efficiency of the cell.

### Electrical properties from current–voltage measurement

The band gap of oyster mushroom particles was calculated using Eq. (3) which was derived from Eq. (2):

3 $${E_g}={\text{ }}hc/\lambda$$

where *E*_*g*_ is the band gap energy, h is the Planck’s constant with the value of 6.626 × 10^− 34^ J/s, c is the speed of light with the value of 3 × 10^8^ m/s, and *λ* is the sample absorbance value.

**Fig. 7:**
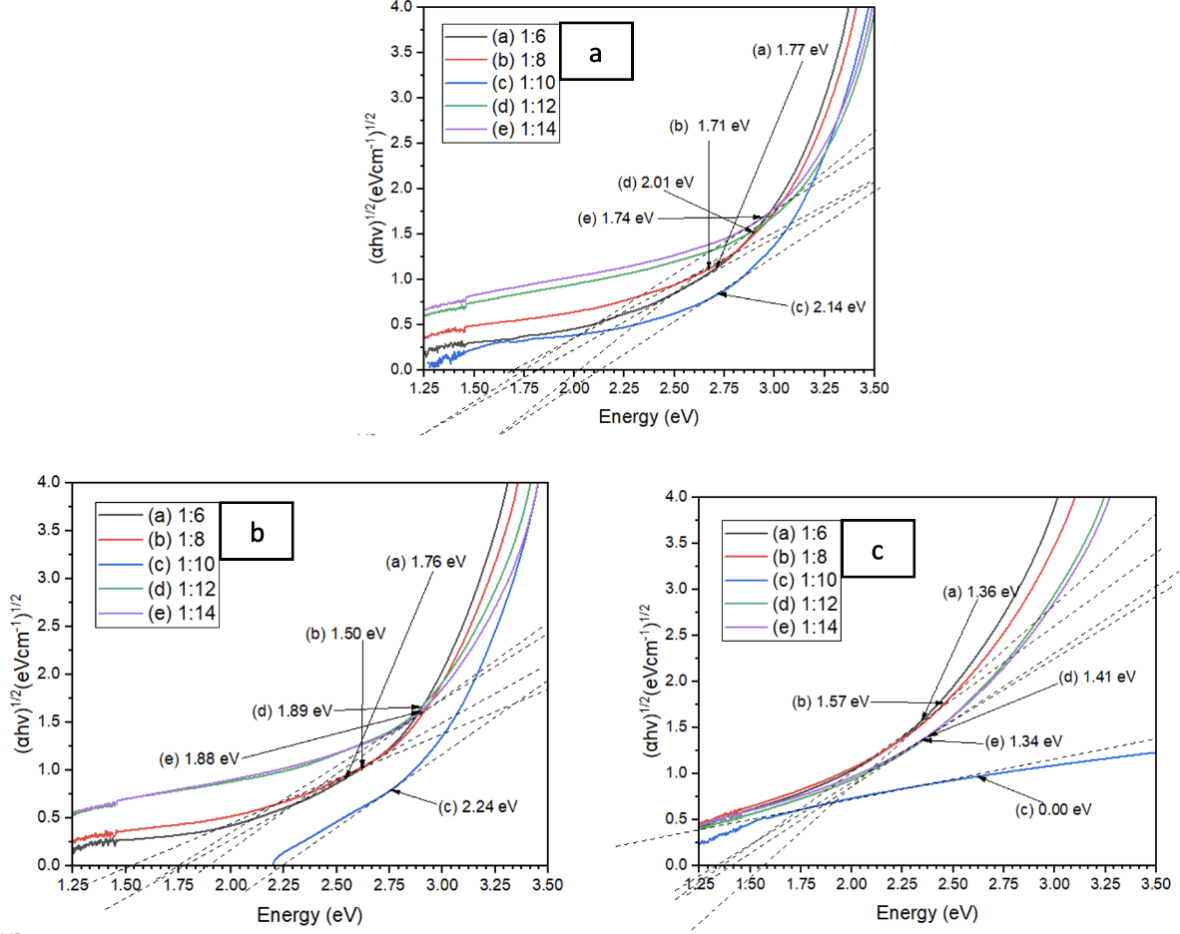
Bandgaps of 1:6, 1:8, 1:10, 1:12 and 1:14 dilution ratio of 3 oyster mushroom samples: (**a**) pink freeze-dried oyster mushroom (PFOM) dye; (**b**) yellow freeze-dried oyster mushroom (YFOM) dye, and (**c**) yellow warm-dried oyster mushroom (YWOM) dye.

The energy gap for the samples is calculated using the graph in Fig. 7. Table 1 shows the value of the energy bandgap which was measured by extrapolating the straight, thin part of the curve to intercept the energy axis at the visible wavelength of 300 to 800 nm. Table 1 depicted the bandgap energy estimated for all 5 different dilution ratios of 3 types of oyster mushroom dyes. From the results obtained, all dilution of PFOM dye with bandgap energy ranging from 1.71 to 2.14 eV, YFOM dye ratio 1:8 (1.50 eV) and YWOM dye ratio 1:8 (1.57 eV) has the optimum bandgap for solar cell application because the ideal band gap for dye sensitizer are roughly around 1.7 to 2.2 eV^30^.

**Table 1:** Energy bandgaps (eV) reading of the oyster mushroom samples.

Samples		Bandgap energy (eV)
Pink freeze-dried oyster mushroom (PFOM)	1:6	1.77
	1:8	1.71
	1:10	2.14
	1:12	2.01
	1:14	1.74
Yellow freeze-dried oyster mushroom (YFOM)	1:6	1.76
	1:8	1.50
	1:10	2.24
	1:12	1.89
	1:14	1.88
Yellow warm-dried oyster mushroom (YWOM)	1:6	1.36
	1:8	1.57
	1:10	0.00
	1:12	1.41
	1:14	1.34

As energy bandgaps widen, electrons with lower excitation energies can become free electrons in a conduction band, increasing the efficiency of the electric solar system (by about 3% for 1 eV). The bandgap of the semiconducting material used has a direct impact on the performance of photovoltaic devices. A bandgap that is too large will not produce a photovoltaic effect, while a band gap that is too small will cause excess energy from photons to heat up the lattice^40^.

Optimal photosensitizer of DSSC must be rapidly regenerated by the mediator layer to avoid electron recombination processes and be fairly stable, both in the ground and excited states. The ideal sensitizer for a photovoltaic cell converting standard air mass (AM) 1.5 sunlight to electricity, must absorb all light below a threshold wavelength of about 900 nm, which is equivalent to a semiconductor with a bandgap of 1.4 eV. Overall the cell performance is subjected to a number of factors but fundamental considerations relating to the dye are how efficiently such as the molecules absorb incident photons, photons are converted to electron–hole pairs and separation and collection occurs^36^.

**Fig. 8:**
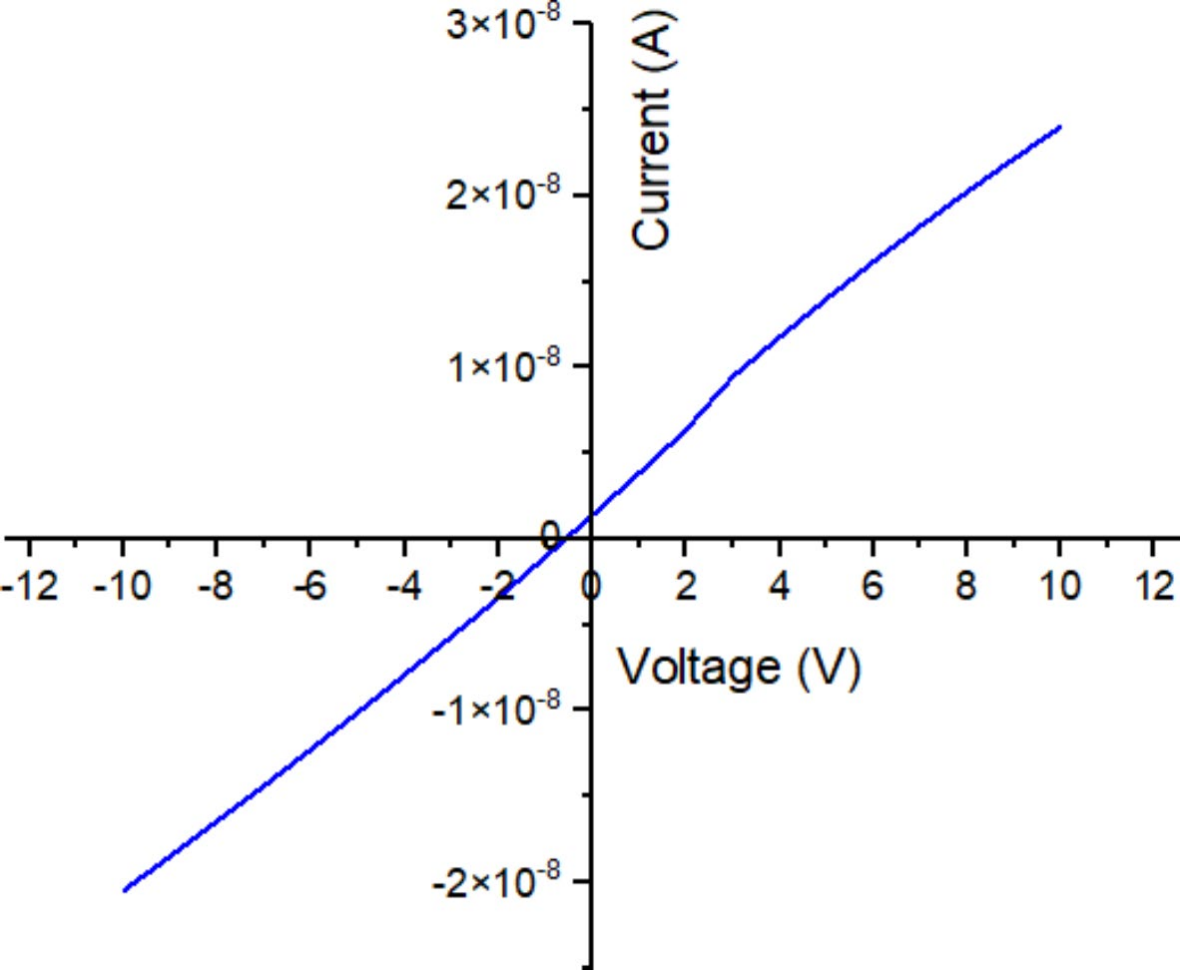
I–V measurement plots of TiO_2_ photoanode after being immersed in pink freeze-dried oyster mushroom dye.

Figure 8 displays the current-voltage (I–V) measurements of TiO_2_ photoanode with pink freeze-dried oyster mushroom (PFOM) dye which being optimized from previous analysis in UV–Vis, FESEM and AFM in this study. It suggests that the TiO_2_ thin film and the Au metal contact have ohmic contact properties because of its symmetrical and linear shape^41^. This result exhibit that not all metal-semiconductor junctions will form a rectifying curve. This condition is called ohmic contact. Rectifying properties depend on the metal’s work function, the bandgap of the intrinsic semiconductor and the type and concentration of dopants in the semiconductor. If the work function of metal is greater than the bandgap of semiconductor material and the concentration of the semiconductor used is more dopant (especially p-type), a linear ohmic contact will occur^42^.

**Fig. 9:**
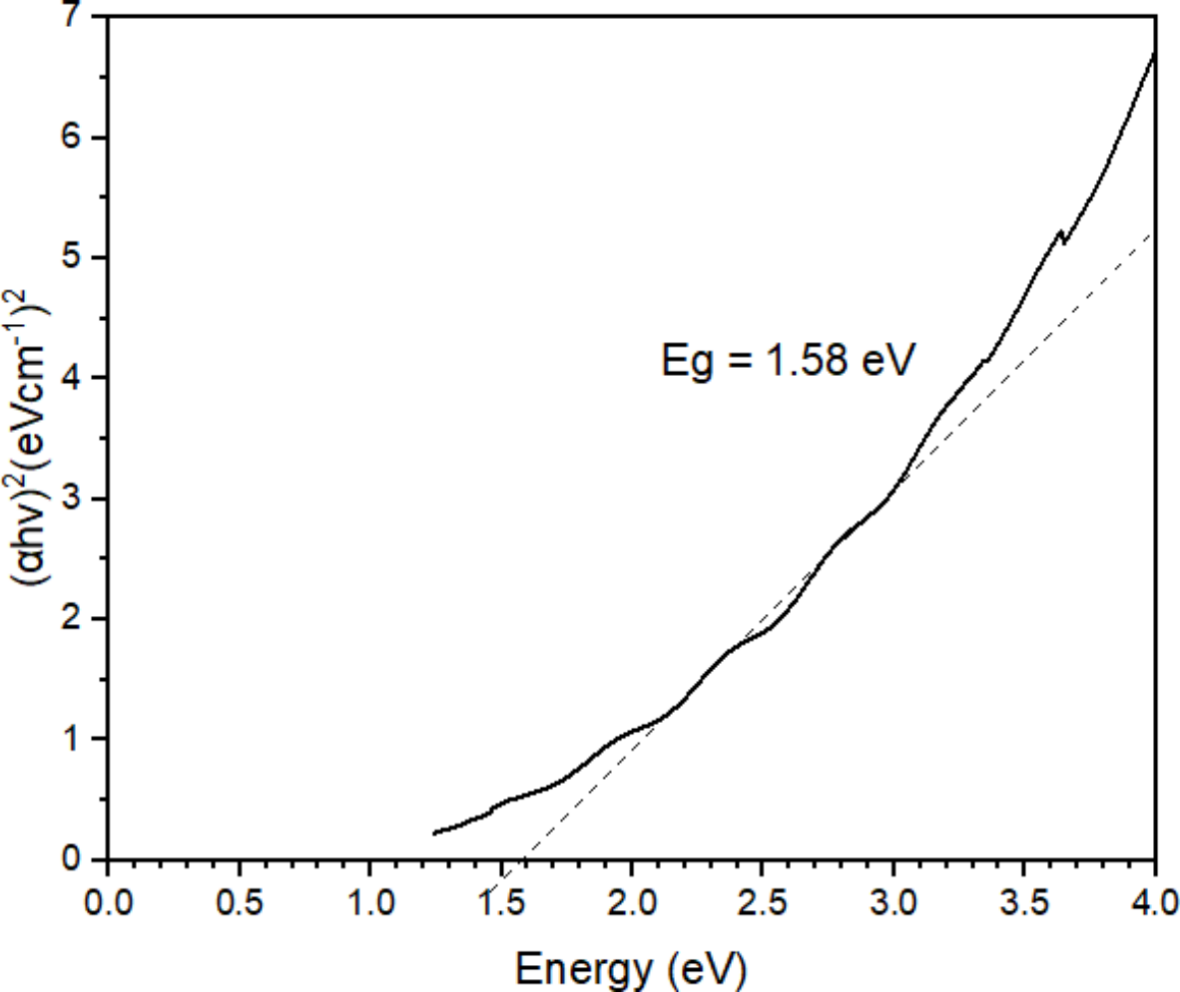
I–V measurement plots of TiO_2_ photoanode after being immersed in freeze-dried pink oyster mushroom dye.

The bandgap energy of the FTO/TiO_2_/PFOM electrode was calculated using the same Eq. (3) and method as mentioned previously. The bandgap energy measured from the Tauc Plot plotted from the absorbance values obtained from UV–Vis analysis (see Fig. 9). The optical bandgap energy calculated is 1.58 eV which is lower than the bandgap of individual PFOM dye (2.14 eV). The dye-TiO_2_ interaction creates excitons, a form of electron-hole pairs that are bonded together. Exciton formation causes the observed increase in the optical bandgap energy.

**Fig. 10:**
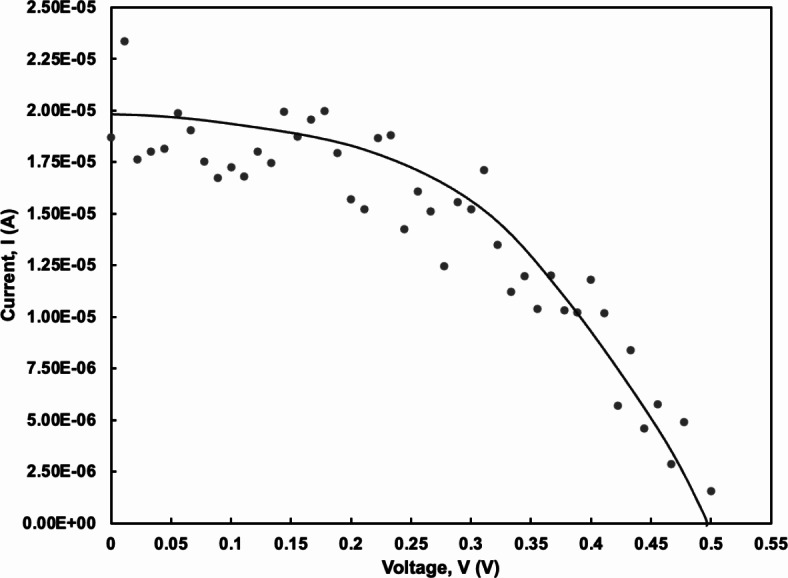
I–V characteristics for fully fabricated DSSC device (FTO/TiO_2_/PFOM/Pt).

The current density-voltage (J–V) characteristics of fabricated DSSC using optimized PFOM dye was characterized using Newport Oriel Sol3A solar simulator under AM 1.5 sun condition (100 mW/cm^2^, 25 °C). Figure 10 shows the I-V characteristics of the DSSC. From the result obtained by the solar simulator, the fabricated FTO/TiO_2_/PFOM/Pt indicated the *J*_*sc*_ of 0.397 mA/cm^2^, *I*_*sc*_ of 1.987 × 10^− 5^ A, *V*_*oc*_ of 0.499 V, *I*_*max*_ of 1.712 × 10^− 5^ A, *V*_*max*_ of 0.3111 V and *P*_*max*_ of 5.327 mW.

Equation (4) was used to calculate the fill factor, *FF* of the device while the power conversion efficiency, *η* is determined by using Eq. (5) explained below:

4 $$\:FF=\frac{({V}_{max}\times\:{I}_{max})}{({V}_{oc}\times\:{I}_{sc})}$$

where *V*_*max*_ and *I*_*max*_ are the maximum value of the voltage and current, *V*_*oc*_ is the open circuit voltage which occurring when the maximum current that happens when the voltage across the device is zero, *I*_*sc*_ is the short circuit current which is the maximum current that exists when the voltage across the device is zero. While the power conversion efficiency, *η* is determined by the following equation with *P*_*inc*_ is the radiation power incident on the cell:

5 $$\:\eta\:=\frac{{V}_{oc}\times\:{I}_{sc}\times\:FF}{{P}_{inc}}$$

Performance of the fabricated device was evaluated from the findings from solar simulator with FF is 0.538 and the overall efficiency of the cell is 0.107%. Table 2 below summarized the results obtained.

**Table 2:** Data of short-circuit current density, open-circuit voltage, fill factor and efficiency of fabricated DSSC.

Short-circuit current, I_sc_ (A)	Open-circuit voltage, V_oc_ (V)	Fill factor, FF	Efficiency, η (%)
1.987 × 10^− 5^	0.499	0.538	0.107

The efficiency of the fabricated DSSC obtained is within the range of the previously fabricated DSSC using another source of fungal dye, *Cortinarius* fungi which reported *J*_*sc*_ =1.79 mA/cm^2^, *V*_*oc*_ = 541 mV, *FF* = 0.65 and *η* = 0.64%^39^. The small efficiency of fabricated device may by resulted by the poor electron injection process and poor light absorption abilities in visible region. Low DSSC efficiency of natural dyes can usually be attributed by factors such as low energy of the dye excited state, quick dye molecule recombination, and the presence of compounds in the extracts that facilitate recombination processes in the cells^39^.

Lower conversion efficiency is due to the absence or reduced bonding to the TiO_2_ and low extinction coefficient value. Hence, higher efficiency of the solar cell is attributed to the effective transfer of electron and enhanced absorption in the visible region of the device. The *J*_*sc*_ and *V*_*oc*_ are the two significant parameters in determining the efficiency of the cell^16^.

### Surface morphology analysis of the oyster mushroom dyes pigments

**Fig. 11:**
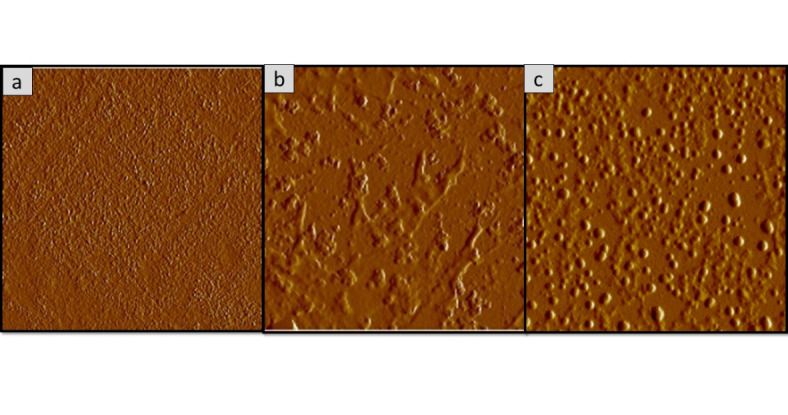
Shows the 2D AFM stereoscopic morphologies of the 3 oyster mushrooms coated with 1 layer on the glass substrate. (**a**) Yellow warm-dried oyster mushroom; (**b**) yellow freeze-dried oyster mushroom, and (**c**) pink freeze-dried oyster mushroom. The scanning area is 10 × 10 μm.

**Fig. 12:**
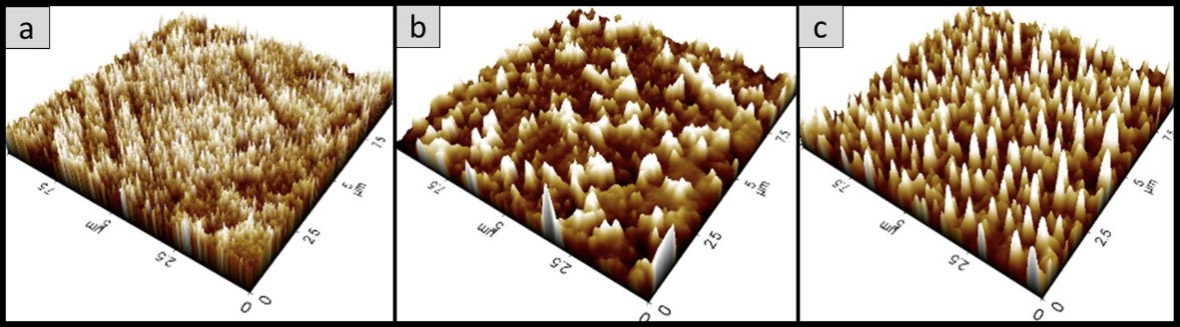
The 3D AFM stereoscopic morphologies of the 3 oyster mushrooms coated with 1 layer on the glass substrate. (**a**) yellow warm-dried oyster mushroom; (**b**) yellow freeze-dried oyster mushroom, and (**c**) pink freeze-dried oyster mushroom. The scanning area is 10 × 10 μm.

**Fig. 13:**
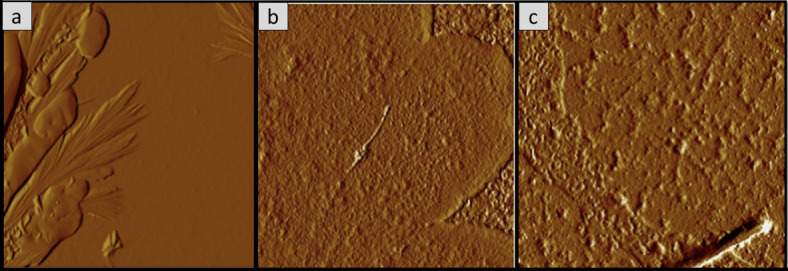
The 2D AFM stereoscopic morphologies of the 3 oyster mushrooms coated with 2 layers on the glass substrate. (**a**) yellow warm-dried oyster mushroom; (**b**) yellow freeze-dried oyster mushroom, (**c**) pink freeze-dried oyster mushroom. The scanning area is 10 × 10 μm.

**Fig. 14:**
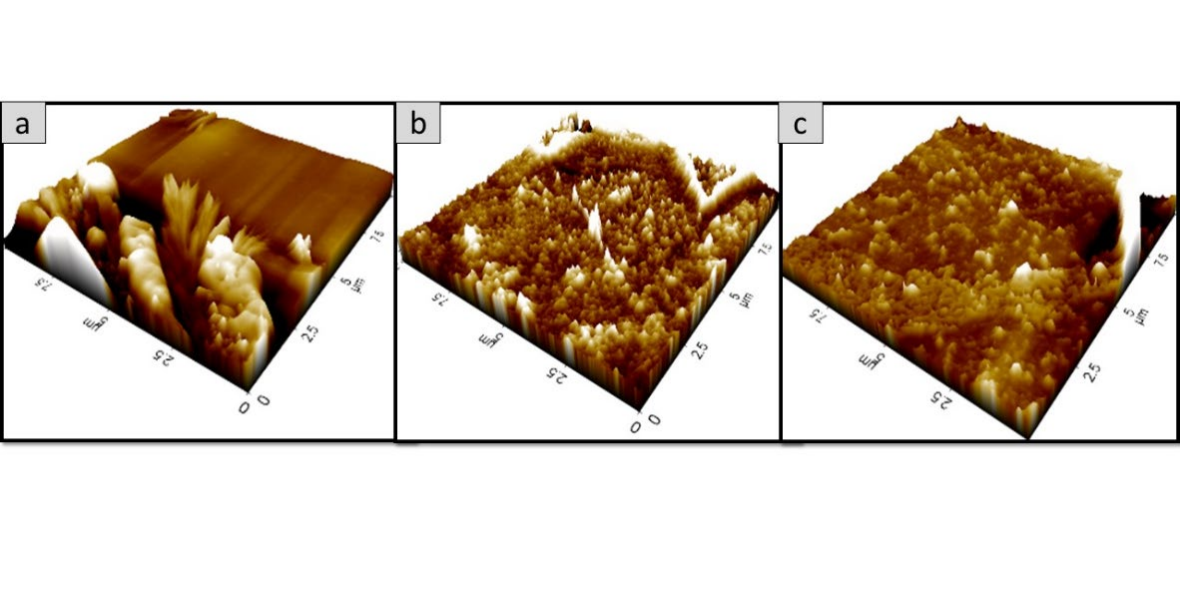
The 3D AFM stereoscopic morphologies of the 3 oyster mushrooms coated with 2 layers on the glass substrate. (**a**) Yellow warm-dried oyster mushroom; (**b**) yellow freeze-dried oyster mushroom, and (**c**) pink freeze-dried oyster mushroom. The scanning area is 10 × 10 μm.

**Fig. 15:**
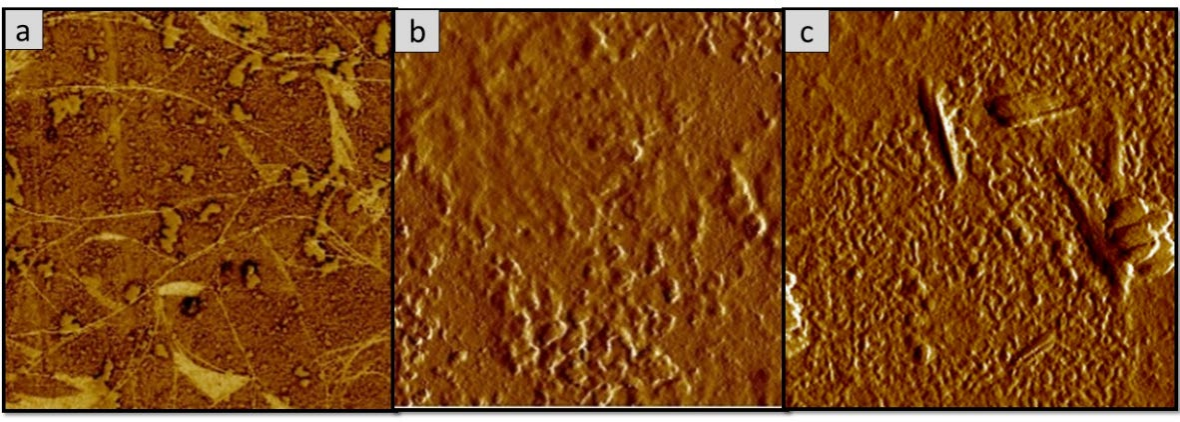
The 2D AFM stereoscopic morphologies of the 3 oyster mushrooms coated with 3 layers on the glass substrate (**a**) yellow warm-dried oyster mushroom; (**b**) yellow freeze-dried oyster mushroom, and (**c**) pink freeze-dried oyster mushroom. The scanning area is 10 × 10 μm.

**Fig. 16:**
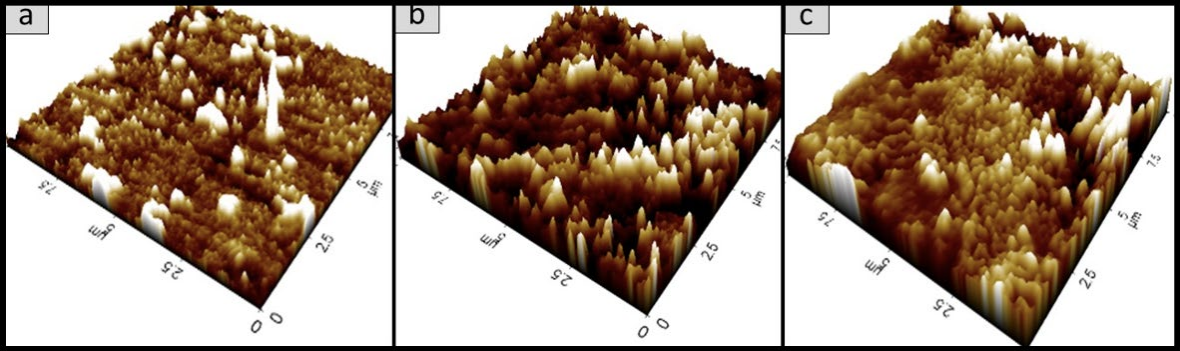
The 3D AFM stereoscopic morphologies of the 3 oyster mushrooms coated with 3 layers on the glass substrate. (**a**) yellow warm-dried oyster mushroom; (**b**) yellow freeze-dried oyster mushroom, and (**c**) pink freeze-dried oyster mushroom. The scanning area is 10 × 10 μm.

Figures 11, 12, 13, 14, 15 and 16 depict the stereoscopic images of thin films with one layer, two layers, and three layers that were scanned by an atomic force microscope. Each thin film’s surface roughness was determined in root mean square (RMS) value, which is a measure that can be derived by AFM, in order to compare the degree of the different layer thin films. The RMS roughness of thin film of 3 different layers of coating for 3 different oyster mushroom dyes are represented in Table 3. This RMS values obtained show that freeze-dried yellow oyster mushroom has higher RMS roughness of thin film compared to the other two oyster mushroom samples. However, for 3 layers, freeze-dried pink oyster mushroom has shown the highest RMS value and its stereoscopic morphologies shows the most compact porous structure. The rough surface of the thin film increases the surface area of the film enabling more adsorption of dye molecule, which consequently increases the absorption of the incident light. As compared to other techniques of coating such as the electrophoretic deposition method and the chemical reduction method, the thin film prepared by the spin coating method tend to possess a high degree of roughness, consistent with the findings in this study. Thus, the porous surface photoanode suggests an enhancement in the adsorption of dye into TiO_2_ structure^43^.

**Table 3:** Root mean square (RMS) measured from AFM analysis of the oyster mushroom samples.

Samples		Root mean square (RMS)
Pink freeze-dried oyster mushroom (PFOM)	1 Layer	15.732
	2 Layer	17.651
	3 Layer	26.922
Yellow freeze-dried oyster mushroom (YFOM)	1 Layer	34.033
	2 Layer	7.121
	3 Layer	20.631
Yellow warm-dried oyster mushroom (YWOM)	1 Layer	2.085
	2 Layer	0.112
	3 Layer	8.808

### Morphological properties

**Fig. 17:**
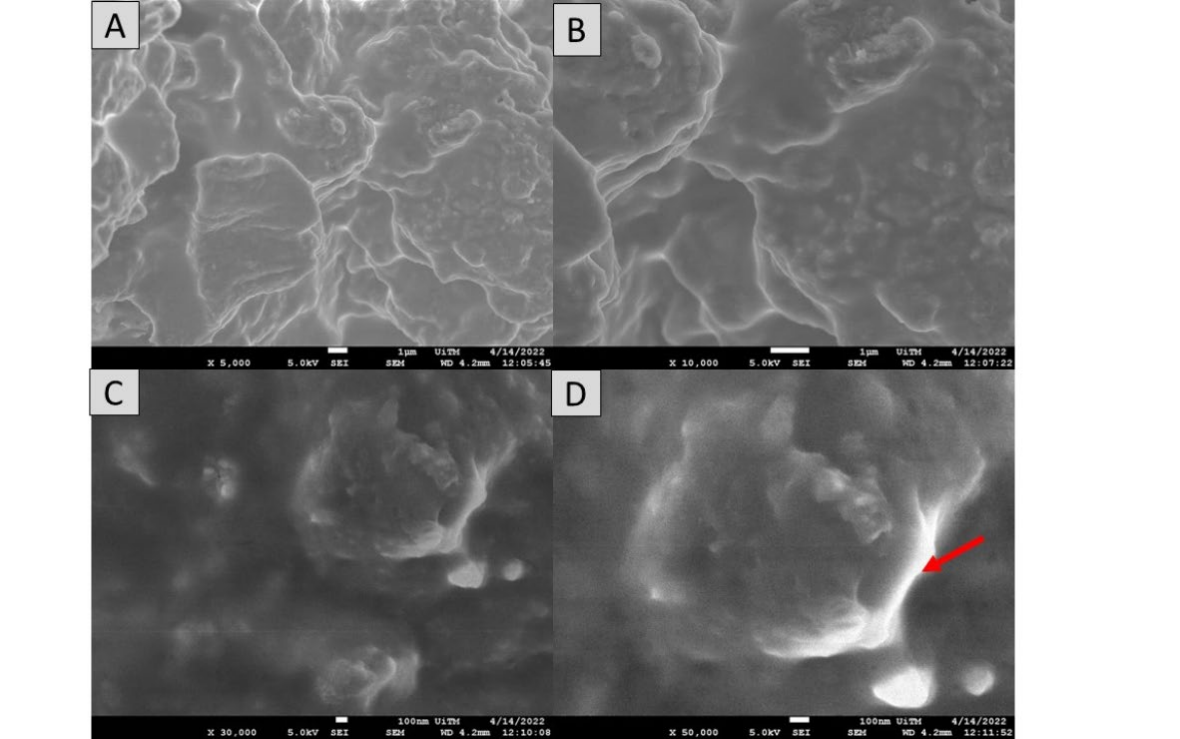
The FESEM images of yellow freeze-dried oyster mushroom. Images A (5000× magnification, bar = 1 μm), Image B (10,000× magnification, bar = 1 μm), Image C (30,000× magnification, bar 100 nm) and Image D (50,000× magnification, bar = 100 nm). Red arrow indicates the presence oyster mushroom pigment.

**Fig. 18:**
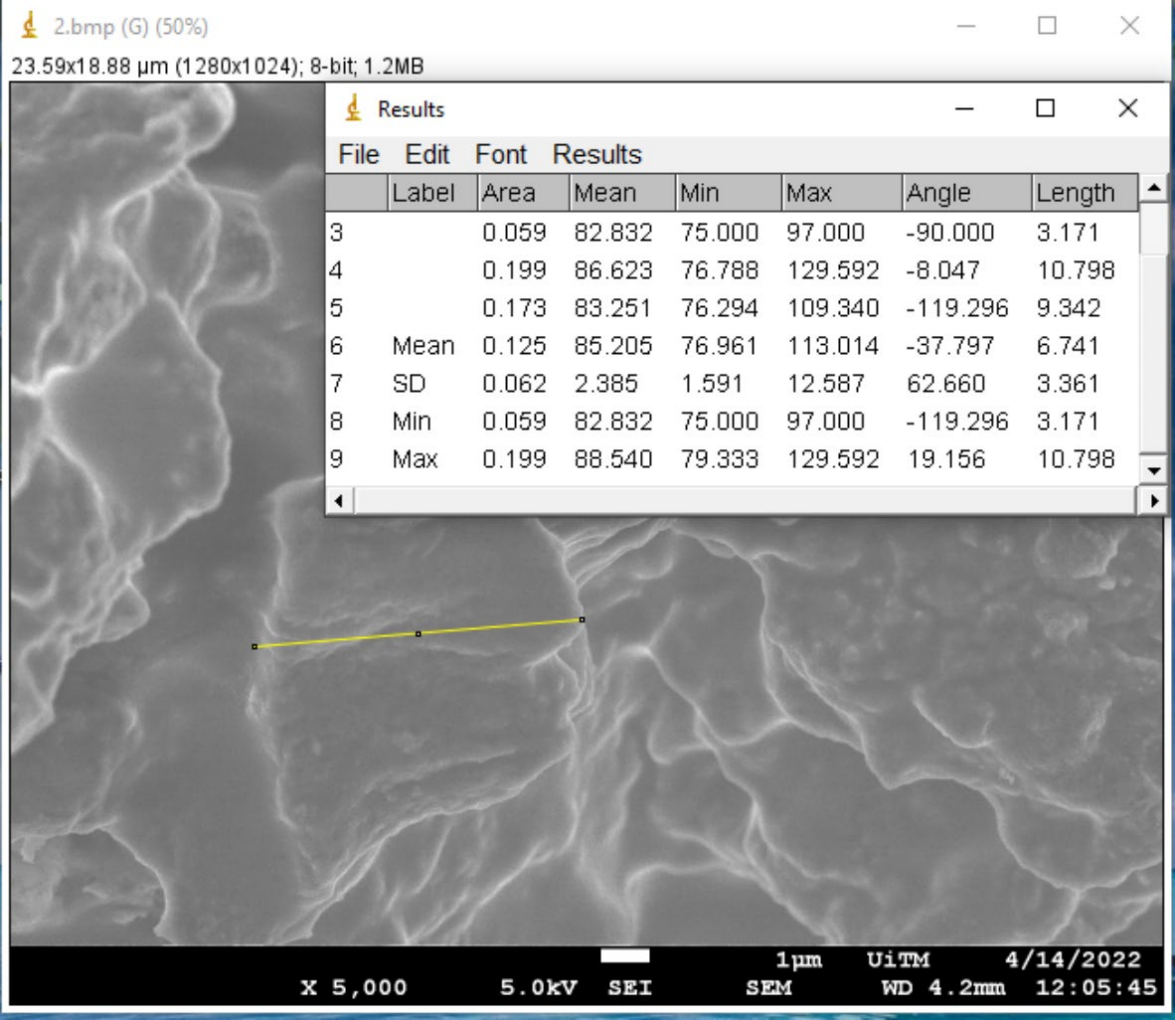
The estimated diameter of mushroom pigment from FESEM images of yellow freeze-dried oyster mushroom in 5000× magnification (bar = 1 μm).

**Fig. 19:**
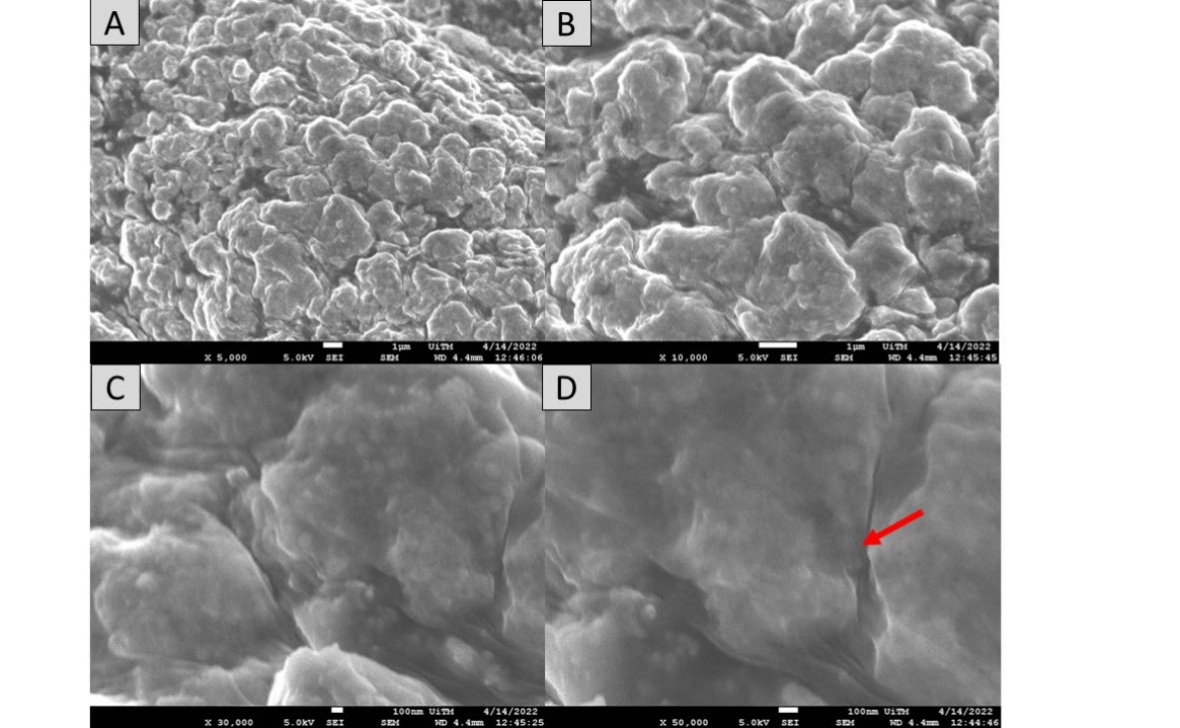
The FESEM images of pink freeze-dried oyster mushroom. Images A (5000× magnification, bar = 1 μm), Image B (10,000× magnification, bar = 1 μm), Image C (30,000× magnification, bar 100 nm) and Image D (50,000× magnification, bar = 100 nm). Red arrow indicates the presence oyster mushroom pigment.

**Fig. 20:**
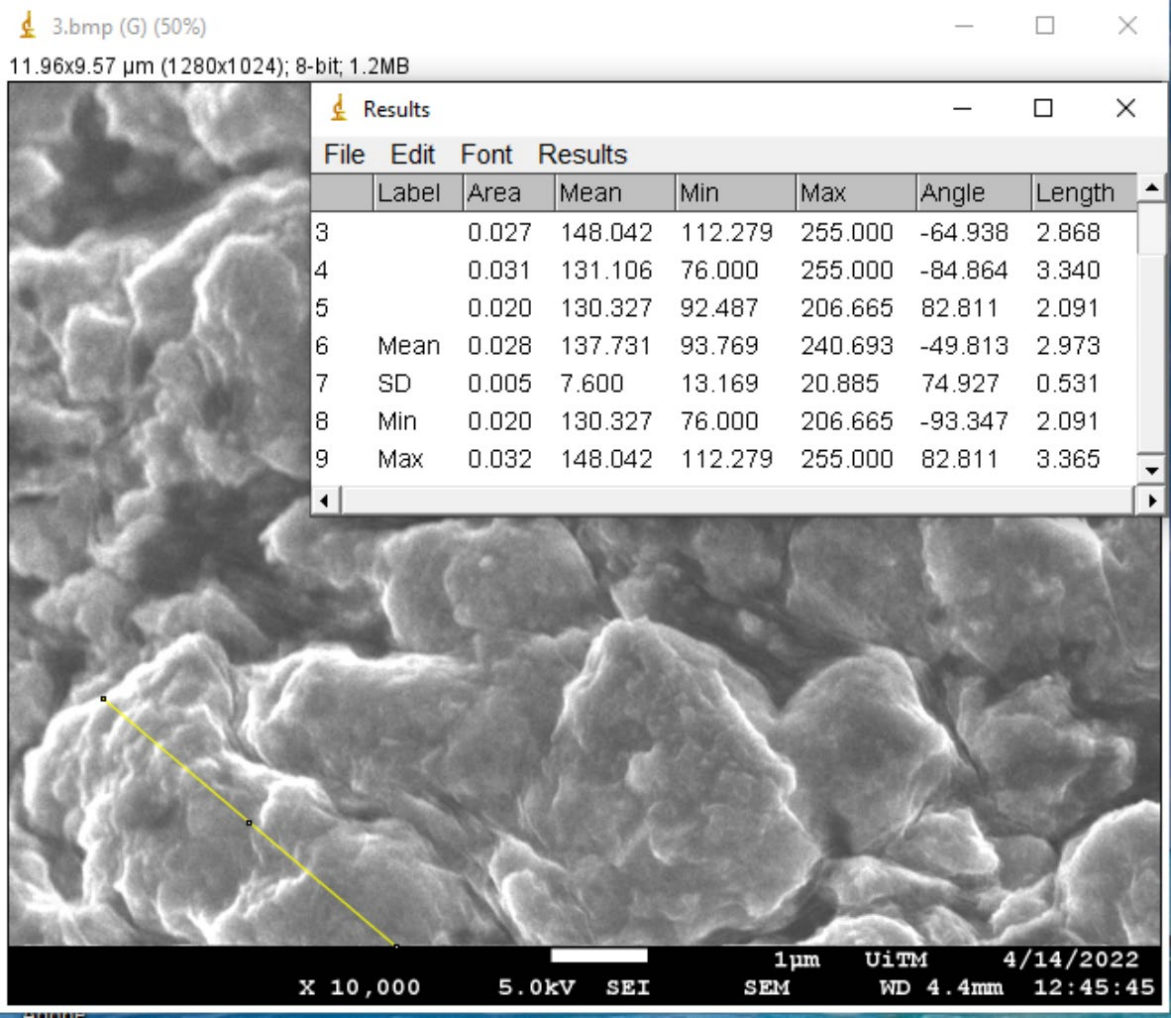
The estimated diameter of mushroom pigment from FESEM images of pink freeze-dried oyster mushroom in 10,000× magnification (bar = 1 μm).

**Fig. 21:**
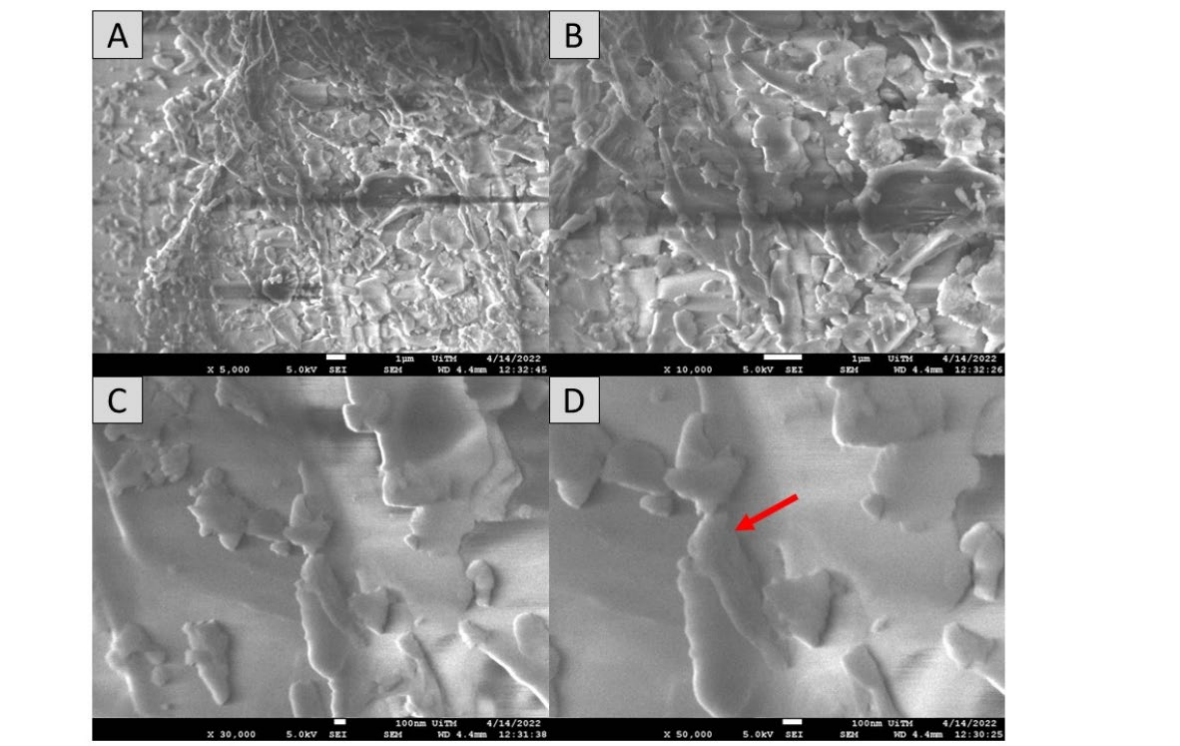
The FESEM images of yellow warm-dried oyster mushroom. Images A (5000× magnification, bar = 1 μm), Image B (10,000× magnification, bar = 1 μm), Image C (30,000× magnification, bar 100 nm) and Image D (50,000× magnification, bar = 100 nm). Red arrow indicates the presence oyster mushroom pigment.

**Fig. 22:**
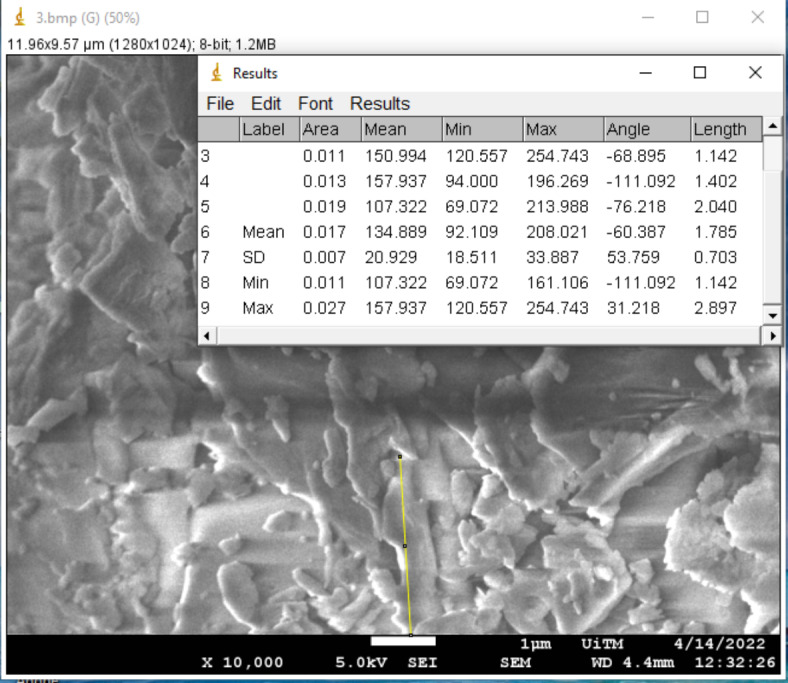
The estimated diameter of mushroom pigment from FESEM images of yellow warm-dried oyster mushroom in 10,000× magnification (bar = 1 μm).

Surface morphology has been demonstrated to have a significant impact on the absorption and charge transport behavior, which impacted the DSSC’s photovoltaic performance. The morphological properties of the pigments in each mushroom dye’s microstructure were examined using Field Emission Scanning Electron Microscope. Figures 17, 18, 19, 20, 21 and 22 shows the microstructure images of yellow freeze-dried oyster mushroom, pink freeze-dried oyster mushroom and yellow warm-dried oyster mushroom dyes powder respectively. The shapes of the mushroom pigment looked to be amorphous solid and irregular^44^. The estimated particle size of yellow freeze-dried oyster mushroom, freeze-dried pink oyster mushroom and warm-dried yellow oyster mushroom measured using Image J Software are 6.741 μm, 2.973 μm and 1.785 μm respectively, and the information is summarized in Table 4. The ultrastructural characteristics of the pigments were observed with FESEM in this study is similar to that of melanin, as reported previously^45^.

By the morphological observation, better light absorption is made possible by the freeze-dry pink oyster mushroom dye morphological characteristics. Additionally, a somewhat bigger grain size lowers the charge transport impediment, improving the photovoltaic performance of DSSCs^46^. DSSCs performance may increase with increasing dye particle size^47^. Enhancing the photoanode’s microstructure is essential to achieving high solar efficiency in distributed solar power systems. The light harvesting efficiency can be greatly increased by the light-scattering effects^48^. Theoretically, melanin pigment is composed of closely packed spherical particles ranging from 40 to 130 nm in diameter, which are found arranged in concentric layers in the cell wall. Early ultrastructural observations in *Agaricus bisporus*,* Fonsecaea pedrosoi*,* Cladosporium carrionii*, and *Hormoconis resinae* suggested compartmentation of fungal melanins into organelles akin to mammalian melanosomes^38^. The aggregation of these simpler units leads to formation of the granular structure that we refer to as melanin granules, which have functional groups that interact with cell-wall and cell-membrane components such as lipids ((CH_2_)_n_, proteins, and polysaccharides (C=C)^38^.

**Table 4:** Average particle size of 3 types of oyster mushroom dyes measured using ImageJ software.

Sample	Average particle size (µm)
Pink freeze-dried oyster mushroom (PFOM)	2.973
Yellow freeze-dried oyster mushroom (YFOM)	6.741
Yellow warm-dried oyster mushroom (YWOM)	1.785

### Comparison with previous studies

Table 5 shows the results obtained from several literatures related to the characterization utilized in the analysis of the mushroom dyes.

**Table 5:** Comparison with the results obtained from previous studies with similar characterization.

Natural dye	Pigment	Absorption ranges/peak absorbance (nm)	Bandgap (eV)	Particle size (nm)	References
*Spinacia oleracea* (spinach)	Chlorophyll	600–700	1.87	–	^11^
*Helianthus annuus* (sunflower)	Betacyanin	294–448	2.71	–	^49^
*Carica papaya* (Papaya leaves)	Chlorophyll	663	1.87	–	^10^
*Auricularia auricula* (jelly ear mushroom)	Melanin	250–300	–	20–400	^32,50^
*Nannochloropsis* (microalgae)	Chlorophyll	220–250	–	5,000	^47^
*Agaricus bisporus* (mushroom)	Melanin	216 and 336	–	–	^4^
*Amaranthus dubius* (red spinach)	Anthocyanin	531	2.34	–	^51^

## Conclusion

This research proposed a novel natural colour for DSSC extracted from two different species of oyster mushroom with different color which are yellow (*P. citrinopileatus*) and pink (*P. djamor*) to identify if fungal pigments can be used as a dye-sensitizer and photon absorber material for DSSC application. Between two drying methods to preserve the long-term stability of oyster mushroom dye, freeze-drying has proven to be a method than warm-drying. Overall, pink and yellow oyster mushroom dyes has shown the presence of melanin pigment. Melanin having peak absorbance at around 300 nm which in the range of visible light. Melanin has the unique ability to absorb almost every wavelength of light and in contrast to most other natural chromophores, has a broadband monotonic absorption spectrum. In term of optimization, pink freeze-dried oyster mushroom of 1:10 ratio with 3 coating layers had shown the optimal properties to be utilized as photosensitizer for DSSC application via characterization using UV–Vis, FTIR, FESEM and AFM with the bandgap energy of 2.14 eV (in the range of ideal solar cell bandgap), presence several O or –OH groups which capable of chelating to the Ti(IV) sites on the TiO_2_ surface, more uniform and compact distribution with somewhat bigger grain size (2.973 μm) which may lower the charge transport impediment, improving the photovoltaic performance of DSSCs. When characterized using solar simulator, the cell has achieved an efficiency of 0.107%, short-circuit current density, *J*_*sc*_ of 0.397 mA/cm^2^, open-circuit voltage, *V*_*oc*_ of 0.499 V and fill factor of 0.538. This research is still a great opportunity in producing clean and inexpensive energy resource as utilizing fungal dyes will be advantageous due to their non-toxic, easy and inexpensive cultivation and extraction process.

## Data Availability

Data sets generated during the current study are available from the corresponding authors on reasonable request.
